# Intracerebral haemorrhage on the acute stroke unit

**DOI:** 10.1007/s00415-019-09663-9

**Published:** 2019-12-09

**Authors:** M. D. Edwards, T. A. T. Hughes

**Affiliations:** Department of Neurology, University Hospital of Wales, Heath Park, Cardiff, CF14 4XN UK

## Introduction

The acute stroke unit can be one of the busiest clinical areas within a modern general hospital. Stroke physicians are faced with important management decisions on a daily basis, but the evidence base available to guide some complex interventions remains inadequate. This month’s journal club examines three papers which attempt to bridge this gap.

The first paper explores the use of Deferoxamine, an iron-chelating agent, and whether this agent can promote recovery in primary intracerebral haemorrhage (ICH). The second paper looks at a common dilemma in patients diagnosed with ICH taking concurrent anti-thrombotic medication and if these medications should be re-started, in particular regarding the risk of further haemorrhage. The last considers optimal timing for commencement of anticoagulation medication in patients with atrial fibrillation who have presented with an ischaemic stroke.

## Deferoxamine mesylate in patients with intracerebral haemorrhage (i-DEF): a multicentre, randomised placebo-controlled, double-blind phase 2 trial

Supportive management is the standard of care in patients with acute ICH on the stroke unit when neurosurgical intervention is not indicated. Iron is released as haemoglobin products are degraded in the aftermath of a bleed and is known to contribute to neuronal injury and promote oxidative stress and inflammation. This was a phase 2 study investigating whether the use of an iron-chelating agent administered soon after presentation would improve clinical outcome. Deferoxamine (32 mg/kg) was administered by intravenous infusion on 3 consecutive days to 145 patients within 24 h of presentation with ICH. 149 patients were given placebo infusions. A good clinical outcome and the primary outcome measure was deemed to be a modified Rankin scale (mRS) score of 0–2 at day 90 following ICH. Secondary outcome measures looked at mRS scores of 0–3 at day 90, but also at day 180. Although this study suggests the use of Deferoxamine is safe in patients with ICH, the change in clinical outcome between the treatment arm and the placebo arm did not exceed their pre-determined futility threshold. As a result, it was deemed that proceeding to a phase 3 trial was inappropriate.

*Comment*. In review of secondary outcomes there was a significant difference with improved clinical outcome (mRS score of 0–2) at 180 days in the treatment arm. Understandably this raises questions about the timing of assessments in stroke patients whose recovery may continue for 6 or even 12 months after presentation. It should also be noted that only 294 patients were entered into the study from 9070 cases that were assessed for eligibility; if only 3.2% of patients with intracerebral haemorrhage were included, an undeniably small proportion of patients, it must limit the relevance of the findings to routine clinical practice. Although efforts were made to balance key covariates when randomising the groups, it does seem significant that the placebo arm contained more patients with thalamic bleeds and intraventricular extension, both of which are likely to be associated with poorer clinical outcomes. The authors recognise this and recommend caution in the interpretation of the data.

Selim et al. (2019) Deferoxamine mesylate in patients with intracerebral haemorrhage (i-DEF): a multicentre, randomised placebo-controlled, double-blind phase 2 trial. Lancet Neurol 18:428–438.

## Effects of antiplatelet therapy after stroke due to intracerebral haemorrhage (RESTART): a randomised, open-label trial

A third of intracerebral haemorrhage occurs in patients already taking antithrombotic agents and it is not always obvious to the treating physician whether to re-start this medication once the patient has passed through the acute phase of ICH. There is understandable reservation due to the perceived risk of recurrent intracerebral haemorrhage, but if patients are taking this medication for previous ischaemic stroke or ischaemic heart disease they may suffer harm if the medication is stopped. The RESTART trial was designed with this important clinical question in mind. It builds on observational studies that had suggested that if antithrombotic therapy is restarted, the risk of occlusive vascular events is lowered without an increase in recurrence of ICH. The patients involved were those on antithrombotic agents who survived the first 24 h after presenting with an ICH for whom the treating clinician was uncertain about restarting their anti-thrombotic medication.

Patients were included if they were taking antiplatelet or anticoagulant therapy at presentation. 268 patients were randomized to start antiplatelet therapy after being diagnosed with ICH (aspirin, clopidogrel or dipyridamole, at a dose of the clinicians choosing) with 269 in the arm that avoided anti-platelet therapy. Primary outcome was risk of recurrent ICH. Secondary outcomes explored how each arm compared in relation to a composite of all major haemorrhagic events (including intracranial and extracranial haemorrhages) and a composite of all major occlusive events (including ischaemic stroke and myocardial infarction). Unexpectedly the group which restarted antiplatelet therapy had a lower incidence of recurrent ICH but this difference narrowly missed statistical significance (*p* = 0.060).

*Comment*. This trial suggests no increased risk of ICH recurrence in patients who take anti-platelet therapy after an ICH and that there may even be a lower risk of ICH. The RESTART collaboration state that the risk of ICH recurrence is outweighed by the benefit of a reduction in vascular occlusive events that comes with antiplatelet therapy; both cerebrovascular and cardiac disease. This study is an important step forward as it is a prospective randomized trial and its results are likely to be more reliable than the current evidence that is based on observational data. An unexpected observation which did not reach statistically significance, was a reduced risk of recurrent ICH with antiplatelet therapy. Possible explanations include the role of arterial thrombosis in ICH, or that some cases labeled as primary intracerebral bleeds are infarcts into which there has been acute secondary haemorrhage.

The authors comment that only 1 in 12 patients who were eligible for the trial were recruited. It is important to note that only cases where the treating physician was unsure about restarting therapy were included. This means a large number of patients (26%) where the decision was clear were excluded and in addition a large proportion of patients were not recruited as they were too unwell. As a result it may be the case that these results are most relevant to a more mildly disabled patient group. However, the authors should be commended for collecting these valuable data in a difficult patient group and addressing a question which previous studies have proved largely inadequate. Lastly the study did not determine an optimum time for re-starting antiplatelet therapy (noting a wide range of 29–146 days); which remains an area which future similar studies could address.

RESTART collaboration (2019) Effects of antiplatelet therapy after stroke due to intracerebral haemorrhage (RESTART): a randomised, open-label trial. Lancet 393:2613–2623.

## Early versus late anticoagulation for ischaemic stroke associated with atrial fibrillation: multicentre cohort study

A common management dilemma on the stroke ward is the timing of anticoagulation in patients with atrial fibrillation (AF) who present with an ischaemic stroke. A high risk of recurrent ischaemic stroke underlines the importance of starting anticoagulation but a number of factors often dissuade clinicians from immediate intervention with the main concern being secondary haemorrhage. Guidelines from the European Society of Cardiology recommend consideration of a number of factors including National Institute of Health Stroke Scales (NIHSS) score and size of infarct, but the data on which this is based lacks evidence.

In this study, Wilson et al. used data from the CROMIS-2 trial to examine 1355 patients with AF in whom anticoagulation was started after presentation with ischaemic stroke. Participants were split into two groups depending on whether they started anticoagulation within 4 days of presentation, or later. Primary outcome was a composite of adverse events including TIA, stroke (haemorrhagic or ischaemic) and death. Although the early intervention group had a lower 90-day event rate this was not significant in secondary analyses when confounders were taken into account, in particular the premorbid modified Rankin scale.

*Comment*. This study was a post hoc analysis of patients recruited into the CROMIS-2 trial. These data are likely to reflect current practice on a modern stroke unit where anticoagulation is initiated earlier in those patients who are less disabled both before and after their stroke, have a smaller infarct in terms of the vascular territory involved and those in whom direct oral anticoagulants are indicated. These are all important factors for risk of future adverse events including further ischaemic events or haemorrhage. In addition, all participants were required to be tolerant of MR scanning restricting some conclusions. Clearly randomised controlled trials in this important area of stroke management are required and the OPTIMAS study will be of relevance in this regard.

Wilson et al. (2019) Early versus late anticoagulation for ischaemic stroke associated with atrial fibrillation: multicentre cohort study. J Neurol Neurosurg Psychiatry 90:320–325.

